# Investigation of Plant Species with Identified Seed Oil Fatty Acids in Chinese Literature and Analysis of Five Unsurveyed Chinese Endemic Species

**DOI:** 10.3389/fpls.2017.00224

**Published:** 2017-02-22

**Authors:** Changsheng Li, Xiaojun Cheng, Qingli Jia, Huan Song, Xiangling Liu, Kai Wang, Cuizhu Zhao, Yansheng Zhang, John Ohlrogge, Meng Zhang

**Affiliations:** ^1^Plant Science Department, College of Agronomy, Northwest A&F UniversityYangling, China; ^2^CAS Key Laboratory of Plant Germplasm Enhancement and Specialty Agriculture, Wuhan Botanical Garden, Chinese Academy of SciencesWuhan, China; ^3^Department of Plant Biology, Michigan State UniversityEast Lansing, MI, USA

**Keywords:** fatty acid, plant species, Chinese endemic plant, Chinese literature, seed oil, ricinoleic acid

## Abstract

Diverse fatty acid structures from different plant species are important renewable resources for industrial raw materials and as liquid fuels with high energy density. Because of its immense geographical and topographical variations, China is a country with enormous diversity of plant species, including large numbers of plants endemic to China. The richness of this resource of species provides a wide range of fatty acids in seeds or other tissues, many of which have been identified by Chinese scientists. However, in the past, most publications describing analysis of these plants were written in Chinese, making access for researchers from other countries difficult. In this study, we investigated reports on seed and fruit oil fatty acids as described in Chinese literature. Six books and more than one thousand papers were collected and the identified fatty acids and relevant plant species were summarized. In total, about 240 fatty acids from almost 1,500 plant species were identified from available Chinese literature. Only about one third of these species were retrieved in the PhyloFAdb and SOFA online databases of plant fatty acids. By referring to a summary of plant species endemic to China, 277 Chinese endemic species from 68 families have been surveyed for seed fatty acids. These account for <2% of total Angiosperm species endemic to China indicating the scope of species yet to be surveyed. To discover additional new fatty acid structures that might benefit society, it is important in the future to study oilseed fatty acids of the many other Chinese endemic plants. As an example, seeds of five unsurveyed species were collected and their fatty acids were analyzed. Ricinoleic acid was detected for the first time in the Salicaceae family.

## Introduction

Traditional fossil resources are becoming more and more limited and concern is growing about how their consumption impacts climate and the environment. One of the feasible resolutions is to exploit alternative sources of industrial raw materials and energy that can be derived from natural biological resources such as plants. On the other hand, a large number of plant species are facing high risks of extinction. Therefore, investigation of plant resources is of importance to both the conservation of plant diversity and the utilization of renewable plant materials.

Triacylglycerol oils (together with proteins and carbohydrates) represent the major constituents of plant seeds and in a large number of species oil is the most abundant form of carbon. Utilization of oils derived from the plant kingdom has increased steadily at a rate of about 5% annually over the last 50 years (Gunstone et al., 2007). Due to increasing population, to rising standards of living, and to reductions in available fertile land, plant oils will be increasingly needed in the future. The consumption of plant oils is expected to approximately double over the next 15 years.

The plant kingdom contains lipids with an incredible variety of structures, including about 400 fatty acids (Matthäus, 2012) and this diversity is most marked among the seed oils of angiosperms (Badami and Patil, 1980). Although some of the fatty acid structures are considered toxic (Downing et al., 1970; Ford et al., 1986; Hamilton and Harper, 1997), a large number could have potential as raw materials for pharmaceutical and chemical productions (Aitzetmüller, 1998). Therefore, vegetable oils could serve as renewable alternatives to petroleum-derived chemicals as well as fossil fuel (Wang, 2006; Carlsson et al., 2011; Vanhercke et al., 2014). An important societal goal for lipid scientists is to integrate their knowledge of oil and fatty acids existing in plant seeds with modern biotechnology and genetic engineering techniques to achieve new industrial applications for plant oils (Carlsson et al., 2011). Systematic collection and analysis of current knowledge of plant oils and continued analysis of oils from un-surveyed species should provide useful information for their further application and as a guide for biotechnology studies of unusual fatty acids.

China is a very large country with enormous variations in geographical and topographical features. The territory of 9.6 million square kilometers stretches across diverse regions, covering the cold, temperate and tropical zones. The topography of China is divided into three main physical macro-regions, namely Eastern China, Xinjiang-Mongolia, and the Tibetan highlands. Generally speaking, altitude descends from the west to the east coast and three types of terrains, mountains, plateaus and hills, constitute a majority of the country's land surface (70 percent). This wide range of geographical conditions provides suitable environments for a great diversity of both plants and animals (Ying, 2001).

The total number of seed plant species identified in China is ~28,700, among which 52% are endemic (15,100 species). These endemic species belong to 1,584 genera and 191 families (Huang et al., 2011). Research regarding Chinese endemic plants has been carried out in recent decades and resulted in publications that cover many aspects, including for example, genetic variation or diversity of some endangered herbs (Qiu et al., 2005; Ni et al., 2006; Ding et al., 2008; Li et al., 2008; Zhou et al., 2010), features and distribution patterns (Yili, 1994; Zhang and Ma, 2008; Huang et al., 2011; López-Pujol et al., 2011), and some specific regional studies (Wang, 1999). According to these investigations, southwest China is the region where Chinese endemic species' richness is concentrated, such as Yunnan and Sichuan province, which include 57% of China's endemic species. Moreover, more than half of Chinese endemic seed plants are considered to be “provincially-local” endemic plants (Huang et al., 2011).

Since the foundation of the People's Republic of China, research regarding the investigation and statistics of the Chinese oil plant resources have been carried out by several institutes (Liangzhi Jia, 1987) and during the period of 1960s–2000s, a large number of books, research articles and reports were published. However, due to a variety of historical reasons, China had been scientifically isolated in some topics for decades and almost all publications covering plant science were written and published in Chinese. Furthermore, data on seed fatty acid composition had been published in a wide range of periodicals many of which can only be accessed in certain libraries. These situations make it difficult for researchers, especially ones from other countries, to access these results.

In order to summarize and share the achievements of Chinese researchers on seed oils, information on Chinese seed oil plants needs to be collected and included in on-line fatty acid database such as SOFA (http://sofa.mri.bund.de; Aitzetmüller et al., 2003; Matthäus, 2012) and PhyloFAdb (https://phylofadb.bch.msu.edu/). In this study, we summarized plant species with identified fatty acid profiles in Chinese literature, identified fatty acids that have not previously been described in plants, and determined basic information on which Chinese endemic seed plants lack fatty acid profiles. This work may provide useful information for researchers who have interests in fatty acids from plant species in China and in chemodiversity in general. In addition, based on this study, future analysis of Chinese endemic oil plants without identified fatty acid profiles may provide valuable information for exploiting and conserving Chinese wild endemic plant resources and gene sources. By referring to our summarized results, we selected five additional plant species as examples without known fatty acid profiles for analysis in this study.

## Materials and methods

### Methods of literature investigation

Digital literature was searched and downloaded from online Chinese databases, such as CNKI (http://www.cnki.net/), WANFANG DATA (http://www.wanfangdata.com.cn/) and CqVip (http://www.cqvip.com/). In some cases the on-line databases of Chinese scientific literature (CNKI, WANFANG DATA and CqVip) can provide access to full text (caj or pdf file) of the original publications for registered users. Literature and papers concerning research on fatty acids of Chinese oil plants were retrieved by searching using “fatty acid composition,” “fatty acid profile,” “fatty acid analysis by GC,” “seed's fatty acid,” “fruit's fatty acid,” “seed's chemical composition,” or “fruit's chemical composition” as keywords (in Chinese). Additionally, since several Chinese periodicals contain a great proportion of plant oil researches, such as *China Oil and Fats, Food Science, and Renewable Energy Resources*, articles or data could be retrieved by manually searching their contents and directories. Similarly, scanned documents of some early periodicals and books were found on the DUXIU database (http://www.duxiu.com/). Utilizing the resources of libraries (e.g. http://lib.nwsuaf.edu.cn/) and platforms selling used books (e.g. http://www.kongfz.com/), related books were also collected. Digital copies of some of these books can be browsed online and downloaded (e.g. http://www.sslibrary.com/) by registered users from Chinese and non-Chinese universities and institutes. Furthermore, translations are available from a number of services that can help interested readers obtain more detailed information from both journal and books published in Chinese.

The Plant List (http://www.theplantlist.org/) is a working list of more than one million scientific plant names and was used to double check the names referred to by the retrieved Chinese literature. The names of plant species were also compared to Flora of China (http://www.eflora.cn/). All names were noted in Table S1 with “Accepted,” “Unresolved,” “From Flora of China,” “From journals,” or “From books” in the collected plant species list. The status “Accepted” indicates that the Latin name is recommended by The Plant List to refer to the species (or to a subspecies, variety or forma). The status “Unresolved” indicates that there is not enough evidence to determine whether the name should be treated as accepted or not, or there were conflicting opinions that could not be readily resolved. For those names which could not be retrieved from The Plant List, the names were referred to the Flora of China and noted as “From Flora of China.” Some remaining plant names were noted as “From journals” and “From books,” which indicated their original source.

In a previous study on Chinese endemic seed plant species by Huang (Huang et al., 2011), a total of 15,103 species from 191 families and 1,584 genera were identified as endemic to China. Combined with the above summary (Table S1), we identified a list of Chinese endemic seed plants with published fatty acid profiles (Table S2). All these plant species and Chinese endemic plants were compared to the plant list in PhyloFAdb to evaluate which species were already included in on-line fatty acid databases and the overlap of information.

The names of fatty acids recorded in Chinese literature vary considerably in formats. In order to provide a consistent nomenclature for comparison to other literature, normalization of Chinese fatty acid names was carried out by referring to Chemical Abstracts nomenclature rules. In addition, the Open Parser for Systematic IUPAC nomenclature (OPSIN, http://opsin.ch.cam.ac.uk/) was used to examine structure models of fatty acids. In a number of cases the position or configuration (*cis* or *trans*) of double bonds is not specified by the Chinese literature we reviewed. Among these, several have quite similar related fatty acids reported in PhyloFAdb and SOFA. However, there was not enough specific information to determine whether they are exactly the same or not. Furthermore, there were a number of fatty acid structures recorded in Chinese literature that are not included in the PhyloFAdb and SOFA databases as of 2016. These represent plant fatty acid structures that may only be recorded in Chinese literature surveyed here (although PhyloFAdb and SOFA are not completely comprehensive).

Because in any large-scale surveys there may be mistakes in identification, we attempted to classify structures that had more reliable structure identification. We considered that if there were multiple reports on an unusual fatty acid, its identification is more credible than fatty acids reported in only a single study. Additionally, if the content of a fatty acid was very low in the seed oil, its identification was considered less reliable. Therefore, two initial criteria were set to further evaluate fatty acids (1) more than one report and (2) a relative percentage of >2% in total fatty acid of seeds. For fatty acids that met these criteria the corresponding original names from journals were examined and the experimental methods and the identified constituents were double-checked. GC-MS and MS methods were considered to provide relatively credible and reliable analysis to determine the occurrence of specific fatty acids. It is important to note that applying these criteria often provides only a preliminary assessment and researchers are encouraged to make their own judgment based on the primary literature. In particular, double bond position and configuration may not have been definitively established. Furthermore, mistakes in identification likely occurred in both the studies surveyed here and in the datasets recorded in PhyloFAdb or SOFA. Therefore, researchers interested in a particular novel structure should carefully review the analytical methods used. In many cases, it may be important to confirm structures by reanalyzing seeds using newer or more extensive methods for structure determination (Spitzer, 1999).

### Plant materials

Fruits of *Poliothyrsis sinensis* Oliv. (Salicaceae), *Sinojackia xylocarpa* Hu (Styracaceae) and *Sinojackia dolichocarpa* C. J. Qi (Styracaceae) were kindly provided by the Wuhan Botanical Garden, Chinese Academy of Sciences (CAS). Fruits of *Sinowilsonia henryi* Hemsl. (Hamamelidaceae) and *Kolkwitzia amabilis* Graebn. (Caprifoliaceae) were collected from the Museum Garden, Northwest A&F University (Shaanxi, China). For comparing GC retention time of ricinoleic acid, seeds of castor (*Ricinus communis* L.) were also collected. Seeds of all samples were carefully separated in the laboratory from other parts of the fruit.

### Oil extraction and fatty acid analysis

To determine the oil content, ~20 mg seeds from each sample were weighed. Seeds were ground quickly and thoroughly in mortars after adding 2.0 mL chloroform: isopropanol (2:1, V/V) and 500 μg 17:0 TAG as internal standard. The mixture in the mortars was transferred into screw-cap test tubes. The mortars were washed twice using 2.0 mL chloroform: isopropanol (2:1, V/V) and the solution was combined to the above tubes. The suspension was shaken and centrifuged. The supernatant was transferred to another tube and dried under flow of nitrogen gas. Four milliliters of 2.5% H_2_SO_4_/MeOH (V/V) were added into the tube and kept at 80°C for 2 h. Two mL of 0.9 wt % NaCl and 2.0 mL hexane were added and vortexed for 2 min. Then for each sample 700 μL supernatants were transferred into GC vials. The FAME were analyzed with a GC-2010 Plus gas chromatograph system (SHIMAZDZU) equipped with a DB23 capillary column (60 m × 0.25 mm, 0.25 μm). The relative fatty acid contents are presented as weight percentage.

GC-MS-QP2010 gas chromatography-mass spectrometry (GC-MS, SHIMAZDZU) equipped with a DB-5MS column (30 m × 0.25 mm, 0.25 μm, Agilent Technologies) was used for analysis of methyl esters of fatty acid in *P. sinensis* Oliv. seed oil. The gas chromatographic conditions were as follows: an initial temperature of 160°C held for 1 min, increasing to 240°C at the rate of 4°C per min and then held for 16 min. The carrier gas was helium with a 1.0 mL /min flow rate and 96.1 kPa. For mass spectrometric analysis by electron ionization (EI), analysis was carried out using EI mode at 90 eV, scanning in the range of 45–500 m/z. Analysis of peaks were carried out by mass spectral library search system of NIST08s.LIB.

## Results and discussion

### Plant species with identified fatty acid profiles in Chinese literature

Six books in Chinese that describe oil plants were collected and examined: *Manual of Chinese Oil Plants* (Institute of Botany of Chinese Academy of Sciences, 1973), *Chinese Oil Plants* (Liangzhi Jia, 1987), *Oil Plants in Sichuan* (Zongying He et al., 1987), *Oil Plants in Henan* (Junpu Zhang and Huaishan, 1996), *Oil Plants in the Northeast and determination of lipid component* (Tingru Zhu et al., 1980), and *Oil Plants in the Northwest* (Northwest Institute of Botany of Chinese Academy of Sciences, 1977). These valuable books were published during 1960s–2000s and reflect the history of seed oil research in China. In 1973, *Manual of Chinese Oil Plants* was compiled by the Institute of Botany, of the Chinese Academy of Sciences and contains description of more than 600 species and seed fatty acid composition of more than 300 species. *Chinese Oil Plants* was published in 1987, which includes substantial efforts of 12 research institutes and is the most authoritative and comprehensive book of Chinese oil plant resources. This book reports on efforts of hundreds of researchers during 6 years to collect materials and analyze the oil-related data of more than 100 families and nearly 1,000 species (which included most data of *Manual of Chinese Oil Plants* together with data from additional plant species). Apart from these nation-wide works, several books about regional oil plants of China were published by regional institutes, such as Northwest Institute of Botany (Chinese Academy of Sciences) and Chengdu Institute of Biology (Chinese Academy of Sciences). Among these regional research materials, *Oil Plants in the Northwest, Oil Plants in the Northeast and determination of lipid component, Oil Plants in Sichuan*, and *Oil Plants in Henan* were analyzed and provided fatty acid profiles of the corresponding regional oil-plants, including 77 sets of data (containing 76 species), 91 sets (containing 91 species), 250 sets (containing 241 species), and 515 sets (containing 515 species), respectively.

Additionally, more than 1,000 research articles (in Chinese) were published in various periodicals and journals. Most of them were retrieved from CNKI, WANFANG DATA, and CqVip, which are the most commonly used and comprehensive Chinese scientific literature databases.

For our study, plant species with identified fatty acid profiles were listed and relevant families were summarized (Table S1 and Table 1). The compiled results provide data for 1,499 species of Chinese oil plants from 145 families. Among these families, there were 42 with at least 10 species with identified fatty acid profiles whereas the number of species with fatty acid analysis are more limited in the other 103 plant families. More specifically, compared with other families, Fabaceae, Rosaceae, Lauraceae, and Brassicaceae contain more species with known fatty acid profiles, with 97, 94, 87, and 60 species, respectively.

**Table 1:** **Summary of plant families and species with identified fatty acid profiles in Chinese literature**.

**Family**	**Number of species**	**Number of Chinese endemic species**	**Family**	**Number of species**	**Number of Chinese endemic species**	**Family**	**Number of species**	**Number of Chinese endemic species**
Achariaceae	3		Cyperaceae	5		Passifloraceae	2	
Actinidiaceae	4	1	Daphniphyllaceae	5		Paulowniaceae	1	
Adoxaceae	18	7	Dilleniaceae	1		Pedaliaceae	1	
Akaniaceae	1		Dioscoreaceae	2		Pentaphylacaceae	3	
Amaranthaceae	20		Dipterocarpaceae	2		Phyllanthaceae	11	3
Amaryllidaceae	5		Elaeagnaceae	9	2	Phytolaccaceae	1	
Anacardiaceae	22	4	Elaeocarpaceae	11	3	Pinaceae	41	13
Annonaceae	10	3	Ericaceae	3		Piperaceae	1	
Apiaceae	18	2	Eucommiaceae	1	1	Pittosporaceae	5	2
Apocynaceae	10	1	Euphorbiaceae	40	3	Plantaginaceae	3	
Aquifoliaceae	8	1	Fagaceae	18	1	Poaceae	11	
Araceae	1	1	Garryaceae	1		Podocarpaceae	3	
Araliaceae	12	3	Gentianaceae	1		Polygalaceae	1	
Arecaceae	6		Ginkgoaceae	1	1	Polygonaceae	4	
Asparagaceae	5		Grossulariaceae	11	3	Portulacaceae	1	
Balsaminaceae	1		Hamamelidaceae	7	3	Primulaceae	4	1
Basellaceae	1		Helwingiaceae	1		Proteaceae	2	
Berberidaceae	9	2	Hydrangeaceae	5		Ranunculaceae	6	2
Betulaceae	16	6	Hypericaceae	1		Rhamnaceae	13	3
Bignoniaceae	3	1	Icacinaceae	5		Rosaceae	94	17
Boraginaceae	10	1	Iridaceae	5		Rubiaceae	8	1
Brassicaceae	60	2	Juglandaceae	12	3	Rutaceae	41	6
Burseraceae	3		Lamiaceae	49	7	Sabiaceae	2	1
Buxaceae	1		Lardizabalaceae	7	1	Salicaceae	3	
Cactaceae	2		Lauraceae	87	34	Santalaceae	5	
Calophyllaceae	1		Fabaceae	97	9	Sapindaceae	38	8
Calycanthaceae	2	1	Linaceae	2		Sapotaceae	11	1
Campanulaceae	4	1	Lythraceae	3		Schisandraceae	8	3
Cannabaceae	18	4	Magnoliaceae	24		Schoepfiaceae	1	
Capparaceae	6		Malvaceae	44	6	Scrophulariaceae	1	1
Caprifoliaceae	6		Melastomataceae	1		Simaroubaceae	4	
Cardiopteridaceae	1		Meliaceae	12		Smilacaceae	3	2
Caricaceae	1		Menispermaceae	4	1	Solanaceae	21	
Caryophyllaceae	5		Moraceae	7		Stachyuraceae	1	1
Celastraceae	33	13	Moringaceae	1		Staphyleaceae	3	
Chloranthaceae	1		Myricaceae	1		Styracaceae	15	9
Cleomaceae	3		Myristicaceae	7		Symplocaceae	7	
Clusiaceae	8	1	Myrtaceae	5	1	Taxaceae	10	6
Colchicaceae	1	1	Nitrariaceae	3	2	Theaceae	59	22
Combretaceae	4		Nyctaginaceae	1		Thymelaeaceae	4	2
Commelinaceae	1		Ochnaceae	1		Trochodendraceae	1	
Compositae	41	2	Olacaceae	2	1	Tropaeolaceae	1	
Connaraceae	1		Oleaceae	22	6	Ulmaceae	12	4
Convolvulaceae	6		Onagraceae	11		Urticaceae	5	1
Coriariaceae	1		Orobanchaceae	1		Vitaceae	10	2
Cornaceae	17	3	Paeoniaceae	8	6	Xanthorrhoeaceae	1	
Crassulaceae	1		Pandaceae	1		Zingiberaceae	1	
Cucurbitaceae	39	6	Papaveraceae	8	2	Zygophyllaceae	1	
Cupressaceae	8	3						

“*Number of Chinese endemic species” indicates the number of seed plants with identified fatty acid profiles which are endemic to China. Complete lists of species names are provided in Tables S1, S2*.

There are no strict standards to give Latin binomial names of plant species in Chinese literature (especially in early papers) and only Chinese names were given in some references, which sometimes results in confusing and complicated names. For instance, sometimes different species were assigned the same name due to the use of synonyms (Xuqi, 1997; Songlin, 2010). In other cases one plant may have different Chinese names (Changhui, 1980; Yufa, 1980), or sometimes one plant has been categorized into different families because different taxonomic systems were used (Liangzhi Jia, 1987; Shuai et al., 2012). To correctly identify plant names as far as possible, in our investigation all names from Chinese literature were referred carefully to The Plant List (http://www.theplantlist.org/) and Flora of China (http://www.eflora.cn/) to provide assessments of each name. As is shown (Table S1), 1,472 different Latin binomial names of plant species could be determined and retrieved in The Plant List. Among them, 1,428 were regarded as “Accepted” while 44 were accounted as “Unresolved” status. For another 27 species' Latin binomial names that are not included in The Plant List, the original sources of the names have been marked as “From Flora of China,” “From journals,” and “From books,” respectively. In order to evaluate the overlap of plant species between our collection list and PhyloFAdb and SOFA, all plant species from Chinese literature were compared with the plant list in these databases. From the list of all 1,499 Chinese plants with FA data collected here, 529 species from Chinese literature are represented in PhyloFAdb and SOFA (names highlighted in bold font in Table S1), which only accounts for about one third of our new dataset. These results indicated that there are almost 1,000 additional plant species from Chinese literature that have not yet been incorporated into the current largest oil seed composition databases.

Although oil research has been carried out for decades, the knowledge and practical application of the oil seed plant species that are endemic to China is still very limited. Huang's investigation on Chinese seed plants indicated that there are 15,103 species of seed plants endemic to China (Huang et al., 2011). By comparison of this list to our data collection (plant species with identified fatty acid profiles in Chinese literature), 277 Chinese endemic seed plant species have been identified with fatty acid profiles. These plants belong to 68 families and 167 genera (Table 1 and Table S2). Among these genera, two families and nineteen genera are considered to be endemic to China and are highlighted in Table S2. Compared with the 15,103 total species of endemic seed plants, these species with identified fatty acid (277) represent only a small percentage (<2%) of plant species endemic to China. Further analysis showed that only 43 out of these 277 plant species are represented in PhyloFAdb and SOFA (highlighted in Table S2). Among these 43 records, 21 species were from three Chinese reports (Jing-Ping et al., 1981, 1983, 1985) and 22 species were from non-Chinese literature. This suggests that a very large number of Chinese endemic seed plants remain to be further studied. Additionally, conservation efforts toward Chinese endemic seed plants need to be widely recognized and encouraged. Based on our analysis, five previously unsurveyed plant species were chosen and their seeds were collected for subsequent fatty acid analysis.

### Fatty acids identified in Chinese literature

Our survey revealed that more than 250 fatty acid structures are recorded in Chinese journals and books. Names of fatty acids identified in Chinese scientific documents are listed in Tables S3, S4. Although a great number of Chinese publications reported fatty acids in plants, the Chinese names of fatty acids were not consistently presented in different reports, which may cause ambiguities for researchers and is inconvenient for utilization of the data. As is common with many analyses in all languages, the incomplete or improper usages include undetermined position or configuration of double bond, different names for expressing the same fatty acid and a lack of consistent or unified standards of nomenclature (Hui-ying, 1991; Qiang-zhong, 2007; Hong-Li et al., 2008; Tao, 2011; as also described in detail by Dijkstra; http://lipidlibrary.aocs.org/History/content.cfm?ItemNumber=40993).

Names of fatty acids recorded in Chinese journals were double checked, and referred to the Chemical Abstracts nomenclature and to OPSIN for structure images to further identify the fatty acids. Some names, such as 10-Eicosenoic acid and 11,14-Octadecadienoic acid, could be retrieved in the PhyloFAdb or SOFA databases, but their names lacked information on, for example, double bond configuration. These are highlighted with gray fill color in Table S3. In addition, seventeen structures recorded as “fatty acids” in Chinese journals (such as Senecioic acid and Tiglic acid) are likely derived from amino acid biosynthesis or other pathways and these are not widely accepted as fatty acids. These “alternative fatty acids” are listed in Table S4.

In contrast to results presented as summaries in the six books, details on fatty acid analysis and methods could be retrieved for more than 220 fatty acids from seeds published in Chinese journals (Tables S3, S4), including both the common and unusual fatty acids. Nearly half of these fatty acids are not represented in the PhyloFAdb or SOFA databases. Many represent double bond positional isomers of more common fatty acids. There are three considerations for further assessment of these data. One is the number of reports (publications). The second is the relative content of an unusual fatty acid. The third is the method of analysis, among which GC-MS and MS are considered to be more reliable. In many cases isomer alternatives may not have been confirmed by definitive methods and researchers should consult the original literature. We considered that identification of a specific fatty acid was more credible when there were multiple reports of its occurrence. We observed there is a correlation between the number of publications reporting a particular unusual fatty acid and whether it appears in the PhyloFAdb/SOFA databases (Table S5). For instance, there are 65 fatty acids that have only one report according to our analysis and 55 of them could not be retrieved in PhyloFAdb or SOFA databases. There may be two possible reasons for this difference. Due to the great diversity of Chinese plant species, many previously unidentified unusual fatty acids may exist in these oilseed plants. Alternatively, there may be mistakes in the identification of these fatty acids during previous experiments and analysis. It may be important for users interested in these fatty acids to carefully review the methodology and in some cases it will be helpful to perform further analysis using newer analytical procedures.

Regarding fatty acids that are not included in PhyloFAdb or SOFA, the experimental methods were further checked and GC-MS and MS data were tentatively regarded as providing more reliable identifications. When combined with the above two criteria (the number of reports and relative content of unusual fatty acid; described in Section “Materials and Methods”), eleven “new” fatty acids and their corresponding data are regarded to be most credible (Table S6). Eight of these represent isomers of more commonly occurring fatty acids. The other 80 structures in Table S6 are considered to be “potentially new” fatty acids which need to be further confirmed. All related plant species, relative contents of these fatty acids and their source references were summarized (Table S6). Overall, these results provide initial leads to encourage lipid researchers for further studies, for example, focusing on some new fatty acids as the targets of genetic engineering. The data described in this study will be added to PhyloFAdb in the near future.

In addition to the results described above, there were 31 fatty acids presented in Chinese oil books but not in the collected reports or journal papers (italics in Table S3). Eight of them were not retrieved in the PhyloFAdb or SOFA databases. Although no detailed method information for the identification of each fatty acid could be retrieved, because the books were edited and published by groups at authoritative institutes, the data are considered to be more reliable than a single report.

### Seed oil content and fatty acid analysis of five unsurveyed Chinese endemic plants

As an initial example of further analysis based on the work described above, we selected five plant species endemic to China that have not previously been analyzed for fatty acids. Two of these are considered rare and endangered. *P. sinensis* Oliv., an ornamental tree, belongs to the Salicaceae family and is a Chinese endemic seed plant genus. *Sinojackia xylocarpa* Hu and *Sinojackia dolichocarpa* C. J. Qi are both included in the Chinese endemic seed plant genus *Sinojackia* Hu. and *Sinowilsonia henryi* Hemsl. is a species of *Sinowilsonia* Hemsl., which is also endemic to China. Similarly, the *Kolkwitzia* Graebn. genus contains a species named *Kolkwitzia amabilis* Graebn. Both *Sinojackia xylocarpa* Hu and *Sinojackia dolichocarpa* C. J. Qi are in the list of rare and endangered plants in China (Information System of Chinese Rare and Endangered Plants, http://rep.iplant.cn/) and both are also considered to be national-key preserved wild plant species (http://www.ethnoecology.org/eflora/View/plant/ZXBWSpecies.aspx).

The oil content as a percentage of dry weight was determined by analysis of total fatty acids of the seeds by referring to a reference internal standard (Table 2). Both *Sinojackia dolichocarpa* C. J. Qi and *Kolkwitzia amabilis* Graebn. seeds contained a relatively high total fatty acid content (>46 wt.%), which might have potential application to be developed as plant oil sources. The oil contents of *Sinojackia xylocarpa* Hu and *Sinowilsonia henryi* were moderate and rather similar, about 37%. The lowest oil content, about 24%, was found in the analysis of *P. sinensis* Oliv (Table 2).

**Table 2:** **Fatty acid contents of seeds from five Chinese endemic species**.

** 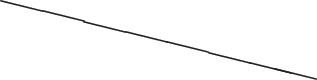 **	***Poliothyrsis sinensis* Oliv**.	***Sinojackia xylocarpa* Hu**	***Sinojackia dolichocarpa* C. J. Qi**	***Sinowilsonia henryi***	***Kolkwitzia amabilis* Graebn**.
16:0	7.32 ± 0.48	7.05 ± 0.17	6.87 ± 0.43	8.24 ± 0.33	5.76 ± 0.03
18:0	2.81 ± 0.15	1.54 ± 0.15	2.03 ± 0.18	3.91 ± 0.15	2.19 ± 0.07
18:1	3.52 ± 0.326	28.97 ± 0.53	25.55 ± 1.44	14.31 ± 1.97	20.65 ± 0.59
18:2	54.01 ± 1.44	61.29 ± 0.63	64.09 ± 1.32	40.31 ± 2.02	69.26 ± 1.50
18:3	1.10 ± 0.17	0.82 ± 0.51	0.57 ± 0.09	31.45 ± 1.96	1.00 ± 0.85
20:0		0.08 ± 0.02	0.22 ± 0.04	0.95 ± 0.20	0.15 ± 0.03
20:1		0.25 ± 0.10	0.35 ± 0.08	0.60 ± 0.11	0.38 ± 0.27
22:0				0.24 ± 0.05	
Undetermined	31.24 ± 1.25^*^		0.32 ± 0.09		0.61 ± 0.38
Total content^**^/wt %	23.513 ± 2.018	37.133 ± 1.504	46.031 ± 1.173	37.703 ± 0.182	47.201 ± 2.542

*All values are expressed as means ± SD (%) of triplicate determinations*.

* *As described in text and Table S7, this fatty acid in P. sinensis was preliminarily identified by GC-MS as ricinoleic acid*.

** *Relative total fatty acid contents (as percent seed dry weight) are indicated as means ± SD (%) of triplicate determinations*.

The fatty acid composition of seed oils from the above five Chinese endemic species were determined by gas chromatography (GC) by reference to the retention time of known fatty acids (Table 2). Linoleic acid (C18:2) was the dominant component, around 40–70%. Oleic acid (C18:1) also accounted for a relatively high percentage (about 20%), except in the *P. sinensis* Oliv. Linolenic acid (C18:3) content of *S. henryi* seeds was 31.4% representing the second largest content whereas in the other four species the seed linolenic content was very low at ~1%.

Unidentified components were <1% except for *P. sinensis* Oliv.'s where initially an unidentified component represented 32 wt% suggesting it may represent a type of unusual fatty acid. The FAME were further analyzed by GC-MS with comparison to the mass spectral library search system of NIST08s. LIB. The fatty acid undetermined by GC matched the spectra of ricinoleic acid, 9-Octadecenoic acid, 12-hydroxy-, (9Z,12R)- (Table S7). Furthermore, the GC retention time of this component was identical to that of ricinoleic acid extracted from castor seeds. Ricinoleic acid is the major component of the seed oil obtained from Castor plant (*R. communis* L., Euphorbiaceae) and occurs at lower levels in seeds of members of a few other plant families (see: https://phylofadb.bch.msu.edu/tree?measure_id=10274) and its biosynthesis and metabolism are extensively studied (Morris, 1967; Borch-Jensen et al., 1997; Meesapyodsuk and Qiu, 2008; Mavraganis et al., 2010; Beopoulos et al., 2014). Thirty-two percent of ricinoleic acid in seeds from *P. sinensis* Oliv. is lower than *R. communis* L. (about 90%) and *Agonandra brasiliensis* (about 45%). *P. sinensis* Oliv. is a member of the Salicaceae family within the order Malpighiales (which also includes Castor). According to PhyloFAdb and SOFA, ricinoleic has not previously been described in the Salicaceae family (five Salicaceae species have been analyzed so far). Thus, *P. sinensis* might be an alternative plant material for research on the evolution and biosynthesis of this unusual fatty acid.

## Conclusion

Approximately 25% of plant orders and 50% of plant families have not yet been analyzed for their fatty acid composition (https://phylofadb.bch.msu.edu/pages/Whats_Missing). Clearly, many new fatty acid structures are yet to be discovered. The goal of this study has been to increase the accessibility of the wealth of data on seed fatty acids that is published in Chinese literature. Through a comprehensive survey of Chinese literature, the compilations of data achieved in this study have identified fatty acid composition data for almost 1,500 plant species, of which 277 are endemic to China. Most of these 277 species, are not recorded in current on-line databases. In addition, a number of fatty acids not previously recognized as components of plant seeds have been identified in these datasets. Taken together, these data in addition to the plant species and fatty acid structures represented in PhyloFAdb and SOFA, provide a guide to identify key branches in plant evolution whose seeds may be most useful for future discovery of novel fatty acid structures. As an example, analysis of five unsurveyed species resulted in the identification of ricinoleic acid for the first time in the Salicaceae family.

## Author contributions

XC and CL performed data investigation and experiments; CL, XC, QJ, XL, KW, HS, CZ and YZ collected and organized the data; CL, JO and MZ designed this research and prepared the manuscript; all authors read and approved the manuscript.

## Funding

This work was funded by the National Natural Science Foundation of China (31270295).

### Conflict of interest statement

The authors declare that the research was conducted in the absence of any commercial or financial relationships that could be construed as a potential conflict of interest.
