# Supplementary material for: Investigation of Plant Species with Identified Seed Oil Fatty Acids in Chinese Literature and Analysis of Five Unsurveyed Chinese Endemic Species
Source: Front Plant Sci. 2017 Feb 22;8:224. doi: 10.3389/fpls.2017.00224 (PMC5320941; doi:10.3389/fpls.2017.00224)
Supplement: Supplementary file 6 [file Table6.docx]

**Table S6.** Plant species and relative contents of “New” and “Potentially new” fatty acids reported in Chinese journals

| No. | Name of Fatty Acid | Plant Species | Relative content (%) | References |
| --- | --- | --- | --- | --- |
| 1 | Decadienoic acid* | *Sapium sebiferum* (L.) Roxb. | 0.38-5.37 | (Lei, 1989;Jin et al., 2010) |
|  |  | *Thlaspi arvense* L. | 2.12 | (Wang, 2012) |
|  |  | *Beilschmiedia tsangii* var. *delicata* (S.K.Lee & Y.T.Wei) J.Li & H.W.Li | 0.70 | (Jüying Cheng, 1981) |
| 2 | Nonanedioic acid* | *Artocarpus heterophyllus* Lam. | 0.15 | (Xing, 2012) |
|  |  | *Mallotus apelta* (Lour.) Müll.Arg. | 0.73-1.16 | (Liu et al., 2009) |
|  |  | *Vitis vinifera* L. | 0.02 | (Wang et al., 2005) |
|  |  | *Rosa roxburghii* Tratt. | 0.705-2.348 | (Shi et al., 2013) |
|  |  | *Camptotheca acuminata* Decne. | 0.09 | (Hu and Yin, 2003) |
|  |  | *Lonicera japonica* Thunb. | 6.02 | (Chen, 2009) |
|  |  | *Solanum melongena* L. | 0.44 | (Tao et al., 2010) |
|  |  | *Datura stramonium* L. | 0.62 | (Zhang et al., 2008) |
|  |  | *Physalis alkekengi* var. *franchetii* (Mast.) Makino | 0.01 | (Liu, 2011) |
|  |  | *Aesculus chinensis* Bunge | 0.03 | (Zhang et al., 2009a) |
|  |  | *Sophora davidii* (Franch.) Pavol. | 0.15 | (San-Qiao et al., 2006) |
|  |  | *Trigonella foenum-graecum* L. | 0.03 | (Wei et al., 2006) |
|  |  | *Scaphium affine* (Mast.) Pierre | 0.51 | (Wang et al., 2003) |
|  |  | *Hovenia acerba* Lindl. | 0.06 | (Cao et al., 2014) |
|  |  | *Staphylea bumalda* DC. | 0.08-0.1 | (Mao et al., 2004) |
|  |  | *Bupleurum falcatum* L. | 3.68 | (Liu et al., 2000) |
|  |  | *Pyrenaria spectabilis* (Champ. ex Benth.) C.Y. Wu & S.X. Yang | 0.98 | (Yin et al., 2014) |
|  |  | *Evodia lenticellata* C.C.Huang | 1.47 | (Zhao et al., 2009) |
|  |  | *Tetradium ruticarpum* (A.Juss.) T.G.Hartley | 5.36 | (Zhao et al., 2009) |
|  |  | *Zanthoxylum* *bungeanum* Maxim. | 0.18-0.49 | (Yang-Min et al., 2010) |
| 3 | 8-Octadecynoic acid* | *Scleropyrum pentandrum* (Dennst.) Mabb. | 27.12-41.68 | (WU Yu, 2015) |
| 4 | 7,10-Octadecadienoic acid* | *Dendropanax confertus* H.L.Li | 6.34 | (Zhu et al., 1998) |
|  |  | *Datura stramonium* L. | 0.67 | (Zhang et al., 2008) |
|  |  | *Astragalus complanatus* Bunge | 0.06 | (Cui et al., 1989) |
|  |  | *Rubus allegheniensis* Porter | 0.17 | (Liu et al., 2011) |
|  |  | *Allium fistulosum* L. | 4.16 | (Jiale, 2009) |
| 5 | 12,15-Octadecadienoic acid* | *Diplopanax stachyanthus* Hand.-Mazz. | 6.20 | (Zhu et al., 1998) |
|  |  | *Fatsia japonica* (Thunb.) Decne. & Planch. | 4.84 | (Zhu et al., 1998) |
|  |  | *Cornus walteri* Wangerin | 6.20 | (Zhu et al., 1998) |
|  |  | *Rubus allegheniensis* Porter | 0.02 | (Liu et al., 2011) |
|  |  | *Paeonia* × *suffruticosa* Andrews | 0.01 | (Qian et al., 2014) |
| 6 | 14,17-Octadecadienoic acid* | *Salicornia europaea* L. | 2.32 | (Liu et al., 2005) |
|  |  | *Parthenocissus quinquefolia* (L.) Planch. | 0.04 | (Wang, 2010) |
|  |  | *Lactuca sativa* L. | 6.34 | (Fang et al., 2012) |
| 7 | 8,11-Octadecadienoic acid* | *Taxus wallichiana* var. *mairei* (Lemée & H.Lév.) L.K.Fu & Nan Li | 11.75 | (Liu et al., 2012a) |
|  |  | *Castanea mollissima* Blume | 45.74 | (Hui et al., 2008) |
|  |  | *Michelia alba* DC. | 21.64 | (Liu and Chen, 2013) |
|  |  | *Magnolia macclurei* (Dandy) Figlar | 59.54 | (Liu and Chen, 2013) |
|  |  | *Leptochloa chinensis* (L.) Nees | 0.03 | (Zhu et al., 2009) |
|  |  | *Kalimeris indica* (L.) Sch.Bip. | 60.56-60.7 | (Jiang et al., 2009) |
|  |  | *Trichosanthes kirilowii* Maxim. | 43.12 | (Yan et al., 2008) |
|  |  | *Solanum melongena* L. | 29.87-62.6 | (Tao et al., 2010) |
|  |  | *Datura stramonium* L. | 4.56 | (Zhang et al., 2008) |
|  |  | *Physalis alkekengi* var. *franchetii* (Mast.) Makino | 0.01 | (Liu, 2011) |
|  |  | *Punica granatum* L. | 2.58 | (Zhao et al., 2005) |
|  |  | *Senna tora* (L.) Roxb. | 41.80 | (Zhang et al., 2006) |
|  |  | *Cicer arietinum* L. | 0.87 | (Gao et al., 2008) |
|  |  | *Astragalus complanatus* Bunge | 7.88 | (Cui et al., 1989) |
|  |  | *Hylocereus undatus* (Haw.) Britton & Rose | 20.25 | (Wang et al., 2012b) |
|  |  | *Rubus allegheniensis* Porter | 0.03 | (Liu et al., 2011) |
|  |  | *Viburnum cylindricum* Buch.-Ham. ex D. Don | 28.24 | (Yang et al., 2009) |
| 8 | 10,13-Octadecadienoic acid* | *Mallotus apelta* (Lour.) Müll.Arg. | 20.28-26.42 | (Liu et al., 2009) |
|  |  | *Kalimeris indica* (L.) Sch.Bip. | 2.11 | (Jiang et al., 2009) |
|  |  | *Rubus vulgaris* Weihe & Nees | 0.04 | (Zhang, 2011) |
|  |  | *Momordica charantia* L. | 0.25 | (Dan et al., 2006) |
|  |  | *Actinidia arguta* (Siebold & Zucc.) Planch. ex Miq. | 0.20 | (Liang et al., 2011) |
|  |  | *Solanum melongena* L. | 0.12 | (Sun et al., 2010;Tao et al., 2010) |
|  |  | *Astragalus complanatus* Bunge | 0.05 | (Cui et al., 1989) |
|  |  | *Trachycarpus fortunei* (Hook.) H.Wendl. | 35.24-37.82 | (Liu et al., 2008) |
|  |  | *Fagopyrum esculentum* Moench | 32.91 | (Fan et al., 2004) |
|  |  | *Prunus pseudocerasus* Lindl. | 0.75-1.58 | (Zheng et al., 2010) |
|  |  | *Mallotus apelta* (Lour.) Müll.Arg. | 23.78 | (Liu, 2008) |
|  |  | *Allium fistulosum* L. | 3.83 | (Jiale, 2009) |
| 9 | 10-Octadecenoic acid* | *Ficus carica* L. | 1.98 | (Qiang et al., 2012) |
|  |  | *Brassica rapa* L. | 1.23-1.49 | (Mahamuti et al., 2012) |
|  |  | *Nepeta tenuifolia* Benth. | 0.075-6.38 | (Yang et al., 2000) |
|  |  | *Perilla frutescens* (L.) Britton | 0.93-1.78 | (Yan et al., 2010) |
|  |  | *Perilla frutescens* var. *crispa* (Thunb.) H.Deane | 0.93 | (Yan et al., 2010) |
|  |  | *Lycium chinense* Mill. | 1.17 | (Shen et al., 2009) |
|  |  | *Sesamum indicum* L. | 12.58 | (Hui et al., 2009) |
|  |  | *Hylocereus undatus* (Haw.) Britton & Rose | 9.70 | (Wang et al., 2012b) |
|  |  | *Camellia sinensis* (L.) Kuntze | 0.09 | (Xinghui et al., 2013) |
|  |  | *Prunus pseudocerasus* Lindl. | 0.95 | (Zheng et al., 2010) |
|  |  | *Arachis hypogaea* L. | 1.09 | (Yan et al., 2014) |
|  |  | *Perilla frutescens* (L.) Britton | 0.93 | (Yan et al., 2014) |
| 10 | 11,13-Eicosadienoic acid* | *Torreya grandis* Fortune ex Lindl. | 3.51 | (Li, 2012) |
|  |  | *Plukenetia volubilis* L. | 0.08 | (Yang et al., 2013) |
|  |  | *Brassica rapa* L. | 0.5-0.73 | (Wang et al., 2011) |
|  |  | *Descurainia sophia* (L.) Webb ex Prantl | 1.44 | (Wang et al., 2011) |
|  |  | *Thlaspi arvense* L. | 2.12 | (Wang et al., 2011) |
|  |  | *Lepidium apetalum* Willd. | 1.24 | (Wang et al., 2011) |
|  |  | *Sinapis alba* L. | 0.51 | (Liu et al., 2001) |
|  |  | *Litsea subcoriacea* Yen C. Yang & P.H. Huang | 4.00 | (Wang et al., 1983) |
|  |  | *Punica granatum* L. | 0.47 | (Zhao et al., 2005) |
|  |  | *Aesculus chinensis* var. *wilsonii* (Rehder) Turland & N.H.Xia | 0.05 | (Chen et al., 2013a) |
|  |  | *Lagerstroemia speciosa* (L.) Pers. | 2.15 | (Zong and Xia, 2004) |
|  |  | *Prunus pseudocerasus* Lindl. | 1.88-2.14 | (Zong and Ma, 2006) |
|  |  | *Nandina domestica* Thunb. | 0.11 | (Wang et al., 2014) |
|  |  | *Schisandra chinensis* (Turcz.) Baill. | 0.22 | (Liu et al., 2014a) |
|  |  | *Allium fistulosum* L. | 0.75 | (Jiale, 2009) |
| 11 | Nonadecanoic acid, 18-methyl-* | *Decaisnea insignis* (Griff.) Hook.f. & Thomson | 6.22 | (Sun et al., 2012a) |
|  |  | *Rubus vulgaris* Weihe & Nees | 2.86 | (Zhang, 2011) |
|  |  | *Rubus idaeu*s L. | 0.98 | (Xin et al., 2011) |
|  |  | *Apium graveolens* L. | 0.09 | (Zhang et al., 2004) |
|  |  | *Morus alba* L. | 0.16-0.3 | (Tian-xi, 2015) |
| 12 | Pentanoic acid, 4-oxo- | *Rosa roxburghii* Tratt. | 1.13 | (Zhang, 2007) |
|  |  | *Prunus armeniaca* L. | 1.04 | (Zhang et al., 2007) |
|  |  | *Seseli condensatum* (L.) Rchb.f. | 1.28 | (Zhang, 2009a) |
| 13 | 3-Heptenoic acid | *Astragalus complanatus* Bunge | 2.01 | (Cui et al., 1989) |
| 14 | Octenoic acid | *Cucurbita moschata* Duchesne | 1.03 | (Xiao Wang, 2002) |
|  |  | *Pyrenaria spectabilis* (Champ. ex Benth.) C.Y. Wu & S.X. Yang | 0.10 | (Yin et al., 2014) |
| 15 | Heptanedioic acid | *Bupleurum falcatum* L. | 0.22 | (Liu et al., 2000) |
| 16 | Octanoic acid, 8-oxo- | *Lonicera japonica* Thunb. | 0.85 | (Chen, 2009) |
|  |  | *Trigonella foenum-graecum* L. | 0.05 | (Wei et al., 2006) |
|  |  | *Pyrenaria spectabilis* (Champ. ex Benth.) C.Y. Wu & S.X. Yang | 0.14 | (Yin et al., 2014) |
| 17 | Octanoic acid, 6-oxo- | *Prunus armeniaca* L. | 0.29 | (Zhang et al., 2007) |
| 18 | Nonanoic acid, 9-oxo- | *Leptochloa chinensis* (L.) Nees | 0.59 | (Zhu et al., 2009) |
|  |  | *Elaeagnus rhamnoides* (L.) A.Nelson | 0.01 | (Hai-Bo and Qin, 2008) |
|  |  | *Trichosanthes kirilowii* Maxim. | 0.02 | (Yan et al., 2008) |
|  |  | *Cucurbita moschata* Duchesne | 0.13 | (Zhang et al., 2003) |
|  |  | *Agastache rugosa* (Fisch. & C.A.Mey.) Kuntze | 0.76 | (Sun et al., 2009) |
|  |  | *Camptotheca acuminata* Decne. | 0.13 | (Hu and Yin, 2003) |
|  |  | *Lonicera japonica* Thunb. | 7.26 | (Chen, 2009) |
|  |  | *Datura stramonium* L. | 1.74 | (Zhang et al., 2008) |
|  |  | *Physalis alkekengi* var. *franchetii* (Mast.) Makino | 0.02 | (Liu, 2011) |
|  |  | *Aesculus chinensis* Bunge | 0.13 | (Zhang et al., 2009a) |
|  |  | *Sophora davidii* (Franch.) Pavol. | 0.27 | (San-Qiao et al., 2006) |
|  |  | *Trigonella foenum-graecum* L. | 0.09 | (Wei et al., 2006) |
|  |  | *Rhodomyrtus tomentosa* (Aiton) Hassk. | 0.02 | (Chen et al., 2008) |
|  |  | *Areca catechu* L. | 0.01 | (Zhang et al., 2009b) |
|  |  | *Staphylea bumalda* DC. | 0.31-0.4 | (Mao et al., 2004) |
|  |  | *Pyrenaria spectabilis* (Champ. ex Benth.) C.Y. Wu & S.X. Yang | 1.36 | (Yin et al., 2014) |
|  |  | *Lepidium apetalum* Willd. | 0.25 | (Wang, 2008) |
| 19 | Nonanoic acid, 7-methyl- | *Rosa roxburghii* Tratt. | 0.09 | (Wang and Chen, 1994) |
| 20 | Decanoic acid, 9-carbonyl- | *Lonicera japonica* Thunb. | 0.45 | (Chen, 2009) |
| 21 | Decanoic acid, 10-oxo- | *Lonicera japonica* Thunb. | 0.53 | (Chen, 2009) |
|  |  | *Tetradium ruticarpum* (A.Juss.) T.G.Hartley | 0.14 | (Zhao et al., 2009) |
| 22 | Cyclopropanenonanoic acid | *Evodia lenticellata* C.C.Huang | 1.10 | (Zhao et al., 2009) |
|  |  | *Tetradium ruticarpum* (A.Juss.) T.G.Hartley | 1.18 | (Zhao et al., 2009) |
| 23 | Undecanoic acid, 10-methyl- | *Nepeta tenuifolia* Benth. | 0.02-0.08 | (Yang et al., 2000;Tianlin, 2001) |
| 24 | Octanedioic acid, 2-hydroxy-, 7-methyl- | *Bupleurum falcatum* L. | 0.25 | (Liu et al., 2000) |
| 25 | Octanoic acid, 6,6-dimethoxy- | *Agastache rugosa* (Fisch. & C.A.Mey.) Kuntze | 3.68 | (Sun et al., 2009) |
| 26 | Undecanedioic acid | *Bupleurum falcatum* L. | 0.14 | (Liu et al., 2000) |
| 27 | Nonanoic acid, 9,9-dimethoxy- | *Vitis vinifera* L. | 0.16 | (Wang et al., 2005) |
| 28 | Tetradectrienoic acid | *Evodia lenticellata* C.C.Huang | 1.87 | (Zhao et al., 2009) |
|  |  | *Tetradium ruticarpum* (A.Juss.) T.G.Hartley | 4.60 | (Zhao et al., 2009) |
| 29 | 8-Tetradecenic acid | *Medicago sativa* L. | 8.10 | (Zhang, 2009b) |
|  |  | *Citrus × aurantium* L. | 2.07 | (Huang and Zhang, 1986) |
|  |  | *Tetradium ruticarpum* (A.Juss.) T.G.Hartley | 0.22 | (Zhao et al., 2009) |
| 30 | 9-Pentadecenoic acid | *Aesculus chinensis* Bunge | 0.15 | (Zhang et al., 2009a) |
|  |  | *Tetradium ruticarpum* (A.Juss.) T.G.Hartley | 0.19 | (Zhao et al., 2009) |
| 31 | Tridecanedioic acid | *Rhus chinensis* Mill. | 0.42 | (ZHANG Jie, 2015b) |
| 32 | Pentadecanoic acid, 9-hydroxy- | *Pyrenaria spectabilis* (Champ. ex Benth.) C.Y. Wu & S.X. Yang | 1.02 | (Yin et al., 2014) |
| 33 | 10-heptadecen-8-ynoic acid, (10E)- | *Hylocereus undatus* (Haw.) Britton & Rose | 1.43 | (Wang et al., 2012b) |
| 34 | 9,12-Heptadecadienoic acid | *Vitis vinifera* L. | 2.02 | (Wang et al., 2005) |
| 35 | 11-Heptadecenoic acid | *Aesculus chinensis* Bunge | 0.33 | (Zhang et al., 2009a) |
| 36 | 11-Hexadecenoic acid, 15-methyl- | *Telosma cordata* (Burm. f.) Merr. | 1.68 | (Yiping, 1998) |
| 37 | Hexadecanoic acid, 9,10-methylene- | *Aesculus chinensis* var. *wilsonii* (Rehder) Turland & N.H.Xia | 0.15 | (Chen et al., 2013a) |
|  |  | *Sesamum indicum* L. | 0.20 | (Hui et al., 2009) |
| 38 | Hexadecanoic acid, 7,8-methylene- | *Calophyllum polyanthum* Wall. ex Choisy | 0.84 | (Zhi, 2005) |
| 39 | 6,12,15-Octadecatrienoic acid | *Abelmoschus moschatus* Medik. | 1.98 | (Jian et al., 2012) |
| 40 | 9,12,16-Octadecatrienoic acid | *Lycium chinense* Mill. | 0.14 | (Shen et al., 2009) |
| 41 | 9,13,16-Octadecatrienoic acid | *Sophora alopecuroides* L. | 3.27 | (Jian Wang, 2002) |
| 42 | 6,9,11-Octadecatrienoic acid | *Lactuca sativa* L. | 0.90 | (Shaoqin et al., 2015) |
| 43 | 11-Octadecynoic acid | *Artemisia carvifolia* Buch.-Ham. ex Roxb. | 0.68-2.02 | (Zheng-Wen et al., 2011) |
| 44 | 10,12-Octadecadienoic acid, (10E,12Z)- | *Morus alba* L. | 0.11 | (Kong, 2013) |
|  |  | *Momordica charantia* L. | 0.27 | (Dan et al., 2006) |
|  |  | *Cornus amomum* subsp. *obliqua* (Raf.) J.S.Wilson | 0.97 | (Li and Huang, 2013) |
| 45 | 13,16-Octadecadienoic acid | *Suaeda corniculata* (C.A.Mey.) Bunge | 0.21 | (Yu et al., 2015) |
|  |  | Citrus grandis (L. ) Osbeck var. *wentanyu* Hort | 0.29 | (Tao et al., 2008) |
|  |  | *Camellia oleifera* Abel | 0.13-0.64 | (Ye et al., 2011) |
| 46 | 6,11-Octadecadienoic acid | *Morus alba* L. | 0.07 | (Kong, 2013) |
| 47 | 10,12-Octadecadienoic acid, (10Z,12E)- | *Momordica charantia* L. | 0.39 | (Dan et al., 2006) |
| 48 | 10,15-Octadecadienoic acid | *Vitis vinifera* L. | 0.18 | (Wang et al., 2005) |
| 49 | 9,13-Octadecadienoic acid | *Vernicia montana* Lour. | 0.41 | (Yu Xue-jian, 1991) |
|  |  | *Hovenia acerba* Lindl. | 1.93 | (Cao et al., 2014) |
| 50 | 11,13-Octadecadienoic acid | *Momordica charantia* L. | 0.88 | (Dan et al., 2006) |
| 51 | 9,15-Octadecadienoic acid | *Allium mongolicum* Regel | 1.57 | (Zhang et al., 2011) |
|  |  | *Paeonia rockii* (S.G.Haw & Lauener) T.Hong & J.J.Li ex D.Y.Hong | 0.03 | (Li-Li et al., 2015) |
| 52 | 9,​17-​Octadecadienoic acid | *Xanthoceras sorbifolium* Bunge | 2.64 | (Chen et al., 2013b) |
| 53 | 16-Octadecenoic acid | *Artocarpus heterophyllus* Lam. | 0.43 | (Xing, 2012) |
|  |  | *Perilla frutescens* (L.) Britton | 1.60 | (Jiao et al., 2008) |
|  |  | *Hylocereus undatus* (Haw.) Britton & Rose | 0.53 | (Jiao et al., 2008) |
|  |  | *Gynostemma pentaphyllum* (Thunb.) Makino | 0.37 | (Liu et al., 2014b) |
| 54 | 4-Octadecenoic acid | *Dendropanax confertus* H.L.Li | 18.41 | (Zhu et al., 1998) |
| 55 | 15-Octadecenoic acid | *Suaeda corniculata* (C.A.Mey.) Bunge | 11.98 | (Yu et al., 2015) |
| 56 | Cyclopentanetridecanoic acid | *Decaisnea insignis* (Griff.) Hook.f. & Thomson | 1.16 | (Sun et al., 2012a;Xiangyu Sun, 2012) |
| 57 | Heptadecanoic acid, 10-methyl- | *Schisandra chinensis* (Turcz.) Baill. | 0.11 | (Liu et al., 2014a) |
| 58 | Heptadecanoic acid, 15-methyl- | *Punica granatum* L. | 0.37 | (Zhao et al., 2005) |
| 59 | Heptadecanoic acid, 14-methyl- | *Pyrus ussuriensis* Maxim. ex Rupr. | 0.12 | (Zhang et al., 2010) |
|  |  | *Mangifera indica* L. | 10.02 | (Ping et al., 2010) |
| 60 | Nonadecadienoic acid | *Arctium lappa* L. | 2.99 | (tian et al., 2002) |
|  |  | *Telosma cordata* (Burm. f.) Merr. | 1.43 | (Yiping, 1998) |
| 61 | 10-Nonadecenoic acid | *Ficus carica* L. | 0.50 | (Qiang et al., 2012) |
|  |  | *Rubus allegheniensis* Porter | 0.32 | (Zhang, 2011) |
|  |  | *Rubus idaeu*s L. | 0.28 | (Xin et al., 2011) |
|  |  | *Trigonella foenum-graecum* L. | 0.04 | (Wei et al., 2006) |
|  |  | *Scaphium affine* (Mast.) Pierre | 1.20 | (Wang et al., 2003) |
|  |  | *Rhus typhina* L. | 0.78 | (Xing et al., 2010) |
|  |  | *Sesamum indicum* L. | 0.05 | (Hui et al., 2009) |
|  |  | *Abelmoschus moschatus* Medik. | 2.43 | (Jian et al., 2012) |
|  |  | *Annona squamosa* L. | <0.3 | (Tang et al., 2012) |
|  |  | *Prunus davidiana* (CarriŠre) Franch. | 0.61 | (Jia et al., 2012) |
|  |  | *Perilla frutescens* (L.) Britton | 0.10 | (Lihui Quan, 1993) |
|  |  | *Schisandra chinensis* (Turcz.) Baill. | 0.16 | (Liu et al., 2014a) |
|  |  | *Rubus allegheniensis* Porter | 0.09 | (Liu et al., 2011) |
|  |  | *Staphylea bumalda* DC. | 0.15-0.21 | (Mao et al., 2004) |
|  |  | *Pyrenaria spectabilis* (Champ. ex Benth.) C.Y. Wu & S.X. Yang | 0.54 | (Yin et al., 2014) |
|  |  | *Prunus persica* (L.) Batsch | 0.36 | (Jia et al., 2012) |
| 62 | 9-Nonadecenoic acid | *Tetradium ruticarpum* (A.Juss.) T.G.Hartley | 0.22 | (Zhao et al., 2009) |
| 63 | 11-Nonadecenoic acid | *Senna tora* (L.) Roxb. | 0.36 | (Zhang et al., 2006) |
|  |  | *Anacardium occidentale* L. | 0.04 | (Sun et al., 2012b) |
| 64 | Octadecanoic acid, 17-methyl- | *Decaisnea insignis* (Griff.) Hook.f. & Thomson | 1.80 | (Sun et al., 2012a;Xiangyu Sun, 2012) |
|  |  | *Akebia trifoliata* (Thunb.) Koidz. | 1.37 | (Xiangyu Sun, 2012) |
|  |  | *Artemisia carvifolia* Buch.-Ham. ex Roxb. | 0.23 | (Zheng-Wen et al., 2011) |
|  |  | *Medicago sativa* L. | 1.08 | (Zhang, 2009b) |
| 65 | Octadecanoic acid, 11-methyl- | *Sesamum indicum* L. | 0.09 | (Hui et al., 2009) |
| 66 | Octadecanoic acid, 16-methyl- | *Lepidium apetalum* Willd. | 0.25 | (Wang, 2008) |
| 67 | Octadecanoic acid, 4-hydroxy- | *Cannabis sativa* L. | 1.09 | (Wen-Feng et al., 2011) |
| 68 | Octadecanoic acid, 6-hydroxy- | *Ziziphus jujuba* var. *spinosa* (Bunge) Hu ex H.F.Chow | 0.74 | (Zhou et al., 2005) |
| 69 | 7,10,13-Eicosatrienoic acid | *Cannabis sativa* L. | 1.75-2.09 | (Wen-Feng et al., 2011) |
|  |  | *Trichosanthes kirilowii* Maxim. | 0.10 | (Yan et al., 2008) |
|  |  | *Datura stramonium* L. | 0.67 | (Zhang et al., 2008) |
| 70 | 11,14,17-Eicosatrienoic acid | *Suaeda maritima* subsp. *salsa* (L.) Soó | 0.22 | (Yu et al., 2015) |
|  |  | *Suaeda corniculata* (C.A.Mey.) Bunge | 0.35 | (Yu et al., 2015) |
|  |  | *Linum usitatissimum* L. | 0.06 | (Ren et al., 2011) |
|  |  | *Jasminum nudiflorum* Lindl. | 16.63 | (Yang et al., 2006) |
|  |  | *Paeonia rockii* (S.G.Haw & Lauener) T.Hong & J.J.Li ex D.Y.Hong | 0.27 | (Li-Li et al., 2015) |
|  |  | *Microula sikkimensis* (C.B. Clarke) Hemsl | 0.04-0.05 | (PI Li, 2013) |
| 71 | Octadecanoic acid, 9,10-methylene- | *Spinacia oleracea* L. | 0.29 | (SUN Feng, 2013) |
| 72 | 6,11-Eicosadienoic acid | *Arctium lappa* L. | 0.29 | (Yuping Ding, 2006) |
|  |  | *Apium graveolens* L. | 0.12 | (Zhang et al., 2004) |
|  |  | *Microula sikkimensis* (C.B. Clarke) Hemsl | 4.78-6.63 | (PI Li, 2013) |
| 73 | 10,13-Eicosadienoic acid | *Torreya grandis* Fortune ex Lindl. | 0.81 | (Li, 2012) |
|  |  | *Chimonanthus praecox* (L.) Link | 0.07 | (Liu et al., 2012b) |
|  |  | *Brassica oleracea* L. | 1.84 | (Liu, 2006) |
|  |  | *Aesculus chinensis* var. *wilsonii* (Rehder) Turland & N.H.Xia | 0.24 | (Chen et al., 2013a) |
|  |  | *Aesculus chinensis* Bunge | 1.04 | (Zhang et al., 2009a) |
|  |  | *Rubus allegheniensis* Porter | 0.51 | (Liu et al., 2011) |
| 74 | 13,16-Eicosadienoic acid | *Citrullus lanatus* (Thunb.) Matsum. & Nakai | 0.26 | (Song et al., 2012) |
|  |  | *Trachycarpus fortunei* (Hook.) H.Wendl. | 1.00 | (Shen, 2012) |
|  |  | *Linum usitatissimum* L. | 0.10 | (Ren et al., 2011) |
| 75 | 9,12-Eicosadienoic acid | *Aesculus chinensis* Bunge | 0.81 | (Zhang et al., 2009a) |
| 76 | 8,11-Eicosadienoic acid | *Parthenocissus quinquefolia* (L.) Planch. | 0.12 | (Wang, 2010) |
| 77 | 7,13-Eicosadienoic acid | *Hevea brasiliensis* (Willd. ex A.Juss.) Müll.Arg. | 9.55 | (Mufeng Ling, 1980) |
| 78 | 10-Nonadecenoic acid, 14-keto- | *Allium cepa* L. | 1.22-1.96 | (Wang et al., 2012a) |
|  |  | *Allium × proliferum* (Moench) Schrad. ex Willd. | 1.29 | (Wang et al., 2012a) |
| 79 | 7-Eicosenoic acid | *Hippophae tibetana* Schltdl. | 0.20 | (Yaonian Xin, 1996) |
|  |  | *Hippophae neurocarpa* S.W. Liu & T.N. He | 0.10 | (Yaonian Xin, 1996) |
|  |  | *Hippophae rhamnoides* subsp. *sinensis* Rousi | 0.20 | (Yaonian Xin, 1996) |
|  |  | *Hippophae rhamnoides* L. Subsp *turkestanica* | 0.10 | (Yaonian Xin, 1996) |
| 80 | Nonadecanoic acid, 11-methyl- | *Hovenia acerba* Lindl. | 1.28 | (Cheng-Ying et al., 2006) |
| 81 | Heneicosatetraenoic acid | *Microula sikkimensis* (C.B. Clarke) Hemsl | 2.22 | (Fu Hua, 1997) |
| 82 | Eicosanoic acid, 18-methyl- | *Akebia trifoliata* (Thunb.) Koidz. | 7.77 | (Xiangyu Sun, 2012) |
| 83 | 9,12-Docosadienoic acid | *Sinapis alba* L. | 0.71 | (Liu et al., 2001) |
|  |  | *Acer truncatum* Bunge | 0.01 | (Wei and Liao, 2011) |
|  |  | *Mucuna sempervirens* Hemsl. | 0.08 | (Ronghan, 1992) |
|  |  | *Mucuna birdwoodiana* Tutcher | 1.10 | (Ronghan, 1992) |
|  |  | *Mucuna hainanensis* Hayata | 2.53 | (Ronghan, 1992) |
|  |  | *Mucuna interrupta* Gagnep. | 1.71 | (Ronghan, 1992) |
|  |  | *Mucuna lamellata* Wilmot-Dear | 0.15 | (Ronghan, 1992) |
|  |  | *Microula sikkimensis* (C.B. Clarke) Hemsl | 0.04-0.07 | (PI Li, 2013) |
|  |  | *Raphanus raphanistrum* subsp. *sativus* (L.) Domin | 0.35 | (ZHANG Jie, 2015a) |
|  |  | *Camellia sinensis* (L.) Kuntze | 0.22 | (Huang et al., 2015) |
| 84 | 13-Docosenoic acid, (13E)- | *Macaranga denticulata* (Blume) Müll.Arg. | 1.50 | (Yu Xue-jian, 1991) |
|  |  | *Sinapis alba* L. | 0.71 | (Liu et al., 2001) |
| 85 | Heneicosanoic acid, 20-methyl- | *Morus alba* L. | 0.03 | (Kong, 2013) |
|  |  | Citrus grandis (L. ) Osbeck var. *wentanyu* Hort | 0.35 | (Tao et al., 2008) |
| 86 | Nonadecandioic acid | *Arctium lappa* L. | 2.99 | (tian et al., 2002) |
|  |  | *Telosma cordata* (Burm. f.) Merr. | 1.43 | (Yiping, 1998) |
| 87 | Octadecanoic acid, 8,10-dimethoxy- | *Zanthoxylum* *bungeanum* Maxim. | 0.65-1.37 | (Yang-Min et al., 2010) |
| 88 | 9-Tricosenoic acid, (9Z)- | *Aesculus chinensis* Bunge | 0.62 | (Zhang et al., 2009a) |
|  |  | *Jasminum nudiflorum* Lindl. | 2.89 | (Yang et al., 2006) |
|  |  | *Ginkgo biloba* L. | 0.20 | (Deng et al., 2007) |
|  |  | *Hippophae neurocarpa* S.W. Liu & T.N. He | 0.50 | (Yaonian Xin, 1996) |
| 89 | Cyclopropanedodecanoic acid, 2-octyl- | *Hylocereus undatus* (Haw.) Britton & Rose | 0.10 | (Wang et al., 2012b) |
| 90 | 13-Tetracosenoic acid | *Descurainia sophia* (L.) Webb ex Prantl | 1.49 | (Li, 2007) |
| 91 | Hentriacontanoic acid | *Toxicodendron succedaneum* (L.) Kuntze | 0.30 | (ZHANG Jie, 2015b) |

Note: * “New” refers to fatty acid that could not be retrieved in PhyloFAdb or SOFA databases (as of Dec. 2016) and with data tentatively considered reliable because 1) it has been reported in more than one study, 2) analytical methods include GC-MS or MS, and 3) it represents at least 2% of total seed fatty acids. “Potentially new” refers to fatty acids reported in Chinese literature but that did not meet all three of these criteria. We note that dicarboxylic acids have not been reported in PhyloFAdb or SOFA. Further study will be needed to determine if these might be related to suberin-like structures associated with seed coats. All references are listed and not all of them are cited in text.

**Reference:**

Cao, L., Qiji, L.I., Xue, Y., Hui, Y.E., Wang, D., and Yang, X. (2014). Constituents and Antioxidant Activity of Fatty Acids from Hovenia dulcis. *Guizhou Agricultural Sciences*.

Chen, D.J. (2009). GC-MS Analysis of Liposoluble Components in Honeysuckle Seeds. *Food Science* 30**,** 321-323.

Chen, G.Y., Shi, Z.H., Li, H.C., Ge, F.H., and Zhan, H.S. (2013a). Supercritical CO2 extraction and component analysis of Aesculus wilsonii seed oil. *Journal of Chinese Medicinal Materials* 36**,** 475-478.

Chen, O., Dong, F.L., Hai-Yuan, M.A., Zheng, S.Q., Ting-Jun, M.A., and Jia, C.X. (2013b). Extraction of Xanthoceras sorbifolia oil and its physical or chemical properties,composition of fatty acid. *Journal of Beijing University of Agriculture*.

Chen, T., Xia, X.K., Hui-Ning, L.U., and Yang, B.L. (2008). GC-MS Determination of Fatty Acids in Fructus rhodomyrti Seeds. *Chemistry & Industry of Forest Products* 28**,** 108-110.

Cheng-Ying, L.U., Lei, H.P., Zhang, M., Huang, Z.C., and Liu, L.P. (2006). Study on the Supercritical CO2 Extraction of Hovenia acerba Seed Fatty Acids. *Food Science* 27**,** 322-325.

Cui, B.L., Lu, Y.R., and Wei, L.X. (1989). Studies on chemical constituents of Astragalus complanatus R. BR. *Yao xue xue bao = Acta pharmaceutica Sinica* 24**,** 189-193.

Dan, W.U., Deng, Z.Y., and Ying-Li, Y.U. (2006). GC determination of bitter melon seeds fatty acids. *Food Science & Technology*.

Deng, Q.C., Zeng, C.M., Tian, B.Q., Chen, C.Y., and Xie, B.J. (2007). Extraction and fatty acid composition of Ginkgo oil. *China Oils & Fats* 32**,** 76-79.

Fan, Z., Song, Q.B., Qiang, G.R., Sun, P.L., Zhu, X.Y., and Chen, D.X. (2004). GC/MS Analysis of Fatty Acid in Fagopyrum Esculentum Seed Oil. *Food Science* 25**,** 267-269.

Fang, X.U., Wang, Q., and Aisa, H.A. (2012). Physicochemical property and fatty acid composition of Lactuca sativa L. seed oil. *China Oils & Fats* 37**,** 72-74.

Fu Hua, W.Q., Zhou Zhiyu (1997). Analysis of Fatty Acids of Seed Oil of *Microula Sikkimensis* Hemsl in Tianzhu by GC/MS. *ACTA AGRESTIA SINCA* 5.

Gao, P., Zhao, S., and Shao, Z. (2008). Fatty acid composition analysis of chickpea oil by GC-MS. *China Oils & Fats*.

Hai-Bo, B.O., and Qin, R. (2008). Comparative Study on Fatty Acid Compositions in Fructus Hippopha Pulp and Seed Oils. *Food Science*.

Hu, J., and Yin, L. (2003). Study on the property of Camptotheca acuminate seed oil and its composition of fatty acid. *Journal of China Agricultural University* 8**,** 30-32.

Huang, B., Wang, X., and Liang, X. (2015). Effects of Tea Cultivars and Oil Extraction Process on Fatty Acid Component in Tea Seed. *Journal of the Chinese Cereals & Oils Association* 30**,** 65-70 and 75.

Huang, M.Q., and Zhang, L.Y. (1986). THE CHEMICAL COMPOSITIONS OF OILS OF MEDICAL SEEDS (OR FRUITS) OF 23 SPECIES. *Guihaia*.

Hui, R.H., Hou, D.Y., Tie-Chun, L.I., and Liu, X.Y. (2008). Analysis of Fatty Acids in Chestnut by GC-MS. *Food Science* 29**,** 541-542.

Hui, R.H., Hou, D.Y., Tie-Chun, L.I., Liu, X.Y., and Yan-Fei, X.U. (2009). Comparison of Fatty Acid Composition of Black Sesame and White Sesame Seeds. *Food Science* 30**,** 333-334.

Jüying Cheng, S.L., Qinghua Lü, Shengqiu Lin (1981). Research on plant oils in Guangxi. *Guihaia* 1**,** 18-31.

Jia, J., Chen, M.J., Xiang, J.I., and Zhang, L.G. (2012). The Content and Composition Analysis of Fatty Acids of Mountain Peach Seeds. *Seed* 31**,** 22-25.

Jiale, Z. (2009). *PHYSIC-CHEMICAL PROPERTIES AND CHEMICAL COMPONENTS OF SEMEN ALLII FISTULOSI OIL EXTRACTED BY SUPERCRITICAL CARBON DIOXIDE.* Master Degree of Medicine, Nanjing Agricultural University.

Jian, L.I., Wang, W., and Sun, X.H. (2012). Analysis of Components in Volatile Oil and Fatty Acid from Seed of Abelmoschus esculentus. *Hubei Agricultural Sciences*.

Jian Wang, H.W., Yuanling Lv (2002). Analysis of inorganic elements and oil fatty acids from seed of *Sophora alopecuroides* L. *Chinese Medicines* 25.

Jiang, X.G., Hou, D.Y., Hui, R.H., Tie-Chun, L.I., Liu, X.Y., and Xiu-Ming, L.I. (2009). Analysis of Fatty Acids in Kalimeris indica Seeds by Gas Chromatography-Mass Spectrometry. *Food Science* 30**,** 425-427.

Jiao, S., Xie, Z., Qian, L.I., Wang, J., and Qing, L.I. (2008). Composition analysis of Fructus Perillae oil and meal. *China Oils & Fats*.

Jin, L., Mingjing, Y.I., Chen, Y., and Chen, Y. (2010). GC-MS analysis of fatty acid components of Sapium sebiferum seeds from five different habitats. *Journal of Hubei University*.

Kong, L.G.D. (2013). GC-MS analysis of fatty acids from *Morus alba* L seed oil. *Shandong Chinese Medicine Magazine* 32.

Lei, C. (1989). PROGRESS OF THE RESEARCH WORK ON THE COMPONENTS OF STILLINGIA OIL. *Journal of Hubei University*.

Li-Li, L.I., Tang, H., Wei, J.M., Wang, S.Q., and University, G.A. (2015). Optimization of chemical leaching extraction process and fatty acid composition of Paeonia rockii seed oil. *Science & Technology of Food Industry*.

Li, D., and Huang, X. (2013). Physicochemical Properties and Fatty Acid Composition of Cornus obliqua Seed Oil. *Journal of Northeast Forestry University* 41**,** 116-117.

Li, H. (2012). Analysis on Physicochemical Properties and Fatty Acid Composition of Torreya grandis Oil. *Journal of the Chinese Cereals & Oils Association*.

Li, L. (2007). *Study on the chromatographic analysisof α-linolenic acid in Descurainia sophia.* Lanzhou University.

Liang, P., Shu-Qian, L.I., Zhang, B., Liu, C.J., and Xin, G. (2011). Fatty Acid Composition in Fruit of Wild Actinidia arguta Sieb.et Zucc. *Food Science* 32**,** 237-239.

Lihui Quan, S.Z., Puzhu Cong (1993). Analysis of fatty acids of seeds from Perill frutescens L Britt var. arguta Benth Hand Mazz and Perill frutescens L Britt. *Chinese Medical Magazine* 28.

Liu, C.H. (2011). Extraction of Physalis alkekengi L. var. franchetii(Mast.) Makin Seed Oil with Supercritical CO_2 and Analysis of Fatty Acids by GC/MS. *Journal of Changchun University*.

Liu, C.J., Zhang, S.Q., and Meng, X.M. (2014a). Analysis of components in oil and volatile oil from seeds of Schisandra chinensis(Turcz.) Bail. *Science & Technology of Food Industry*.

Liu, J., and Chen, J.F. (2013). Extraction of seed oil and fatty acid analysis from four species in Magnoliaceae. *Guihaia*.

Liu, L. (2006). Analyzing Fatty Acid Composition of Cabbage Mustard Seed Oil by GC-MS. *Journal of the Chinese Cereals & Oils Association* 21**,** 177-179.

Liu, Q., Zhan, L.I., Xie, M., and Zhu, Y. (2001). Component Analysis of Fatty Acids in Mustard Seed. *Journal of Instrumental Analysis*.

Liu, S.B., Chen, P.P., Chun-Hua, Y.I., Liu, Z.X., Cai, S.J., and Chen, G.X. (2008). Supercritical CO2 Fluid Extraction of Trachycarpus fortunei Seed Oil and GC-MS Analysis. *Food Science*.

Liu, S.B., Peng, X.L., Yi, C.H., Chen, P.P., and Zhang, S.X. (2009). Extraction and analysis of kernel oil of Mallotus apelta. *Guihaia*.

Liu, S.B., Tan, X.M., Peng, X.L., Yang, P., and Jiang-Ming, L. (2014b). Extraction,composition analysis and acute toxicity test of seed oil of Gynostemma pentaphyllum. *Guihaia*.

Liu, S.B., Tang, K.H., Peng, X.J., and Ping, H.E. (2012a). Nutritional Components of Taxus chinensis var. mairei Seeds and Acute Toxicity of Its Aril. *Food Science* 33**,** 298-301.

Liu, X., Huang, K., Zhou, J., Zhu, J., and Meng, L. (2011). Supercritical CO2 fluid extraction technology and fatty acid composition of blackberry seed oil. *Transactions of the Chinese Society of Agricultural Engineering* 27**,** 312-315.

Liu, X., Li, M., Wang, H., and Yang, C. (2000). Chemical constituents analysis on the seeds of Bupleurum Falcatum L. *Chinese Journal of Analytical Chemistry* 28**,** 1083-1084.

Liu, X.G., Xia, Y.G., Wang, F., Sun, M., Jin, Z.J., and Wang, G.T. (2005). Analysis of Fatty Acid Compositions of Salicornia Europaea L. Seed Oil. *Food Science*.

Liu, Z. (2008). *Study of Extraction Methods and Analysis of Seed Oil and Pericarp Essential Oil from Mallotus apelta.* Master, Hunan Agricultural University (HUNAU).

Liu, Z.X., Wang, H., Xiang, F., Jian-Wu, H.E., and Chen, G.X. (2012b). Optimization of Extraction Process and GC-MS Analysis for Fat Acid in Chimonanthus praecox Seeds. *Hunan Agricultural Sciences*.

Mahamuti, Milibanhuojia, and Baihetinure (2012). Determination of oil Compositions in Brassica rapa L. seed by Gas Chromatography-Mass. *Journal of Xinjiang Normal University*.

Mao, D.B., Jia, C.X., Jin, B.Q., and Zhang, J.S. (2004). Analysis of fatty acids in staphylea bumalda DC seed oil by GC-MS. *China Oils & Fats*.

Mufeng Ling, G.M. (1980). Study on the seed oil from Brazilian rubber. *World Tropical Agriculture Information* 1**,** 38-41.

Pi Li, H.F., Hu Feng-Zu, Han Tao, Li Yi-Kang, Cheng Da-Zhi (2013). Analysis of the Nutritional Components of *Microula sikkimensis* Seeds. *Acta Nutrimenta Sinica* 35.

Ping, D.U., Yang, F., and Zhang, X.J. (2010). Analysis of Fatty Acid Composition of the Seed Kernel of Mango from Yunnan. *Food Science*.

Qian, M.Y., Mei-Qing, L.I., You, W.U., and Sun, Q. (2014). Analysis of Physicochemical Properties and Fatty Acids of Paeonia ostii Seed Oil by GC-MS. *Natural Product Research & Development* 26**,** 380-383.

Qiang, L.M., Yuan, S.Q., Han, L., Kou, T.S., Zhang, M., and Zhang, Z.D. (2012). Analysis of supercritical carbon dioxide extraction of fatty acids from Ficus carica L.by GC-MS. *Science & Technology of Food Industry* 33**,** 288-291.

Ren, H.W., Xue, L.I., and Tang, X.H. (2011). Characteristic analysis and nutrition evaluation of flaxseed and its oil. *Science & Technology of Food Industry* 32**,** 143-145.

Ronghan, Y.X.a.Z. (1992). Studies on Chemotaxonomy of Mucuna and Stizolobium. *Journal of China Pharmaceutical University* 23**,** 74-76.

San-Qiao, W.U., Xin-Sheng, L.I., and Jiang-Hai (2006). Study on Fatty Acids Composition of Sophora Viciifolia Seed Oil. *Food Science* 27**,** 626-628.

Shaoqin, H.E., Zhang, J., Abulimityili, Hang, B.A., and Hajiakberaisa (2015). Supercritical CO2 extraction of Lactuca sativa L.seed oil and its fatty acid composition. *China Oils & Fats*.

Shen, D. (2012). Analysis of Fatty Acid Composition in Oil Palm Fruit by Chromatography-Mass Spectrometry. *Journal of the Chinese Cereals & Oils Association*.

Shen, H.L., Xiang, N.J., Gao, Q., Chao-Min, N.I., and Miao, M.M. (2009). Analysis of Fatty Acids Components of Medlar by GC/MS. *Journal of Chinese Mass Spectrometry Society* 30**,** 99-104.

Shi, Y.N., Wang, D.P., Lin, M.A., You, Z.F., and Yang, X.S. (2013). Constituents and Antioxidant Activity of Fatty Acids in Seed Oil of Rosa roxburghii. *Journal of Mountain Agriculture & Biology*.

Song, Y.F., Liu, C.H., Wen-Zhong, H.U., Jiang, A.L., and Tian, M.X. (2012). Study on ultrasonic-assisted extraction of seeding-watermelon seeds oil and analysis of fatty acid compositions. *Science & Technology of Food Industry* 33**,** 255-258.

Sun Feng, S.X., Mounir Alim, Wang Chen-Hui, Wang Qiang (2013). Analysis of Fatty Acids of Spinacia Oleracea L. Seeds Oil from XinJiang by Using GC-MS. *Food Research And Development* 34.

Sun, L.Y., Tao, J., Luo, W.W., Hai, C.M., and Tie-Chun, L.I. (2010). Analysis of Fatty Acids in Seed of Eggplant by GC-MS. *Journal of Anshan Normal University*.

Sun, X.L., Jia, C.X., Mao, D.B., Niu, J.P., and Shen, J.C. (2009). Analysis of fatty acids in Agastache rugosa seed by supercritical CO2 extraction and GC-MS method. *Journal of Zhengzhou University of Light Industry*.

Sun, X.Y., Duan, A.L., Gao, G.T., Yan, B., Liu, J.R., and Wen-Juan, M.A. (2012a). Optimization of Decaisnea Insignis seed oil extraction process and analysis of fatty acid. *Science & Technology of Food Industry* 33**,** 236-241.

Sun, Y.F., Yang, K.B., Liu, Y., Liu, Z., and Fang-Liang, L.I. (2012b). Analysis on Fatty Acids in Seeds of Anacardium occidentale L. by GC-MS. *Heilongjiang Agricultural Sciences*.

Tang, D., Zhao, T., Zou, Y., and Yang, L.Q. (2012). Extraction and GC-MS Analysis of Fatty Acids from Annona squamosa L. Seeds. *Food Science* 33**,** 286-289.

Tao, J., Wang, X.C., Tie-Chun, L.I., Hui, R.H., and Hou, D.Y. (2010). Determinating Fatty Acid ingredient in Eggplant Seeds by GC/MS. *Seed* 29**,** 99-101.

Tao, J., Zhang, J.L., Tie-Chun, L.I., Hui, R.H., and Hou, D. (2008). Analysis on Fatty Acids in seed of Citrus grandis(L.) Osbeck var.Wentanyu Hort by GC/MS. *Journal of Anshan Normal University*.

Tian-Xi, L.W.C.L.W.Z.-M.G.D.-Y.J. (2015). Analysis of Fatty Acid Compositions of Mulberry Seed Oil in Three Different Regions. *Food Research And Development* 36.

Tian, W.C., Jie, Z.X., Zeng, L.F., and Ge, C.C. (2002). Analysis of fatty acid in Arctium lappa L. seed oil by GC-MS. *Journal of Plant Resources & Environment*.

Tianlin, Y. (2001). GC-MS analysis of Fatty acid in Foeniculum Vulgare Mill . Seeds using different solvents. *Journal of Shaanxi Normal University(Natural Science Edition)* 29.

Wang, C. (2012). Analysis of Physicochemical Properties and Fatty Acid Composition of Penny Cress Seed Oil. *Journal of the Chinese Cereals & Oils Association* 27**,** 67-70.

Wang, C., Feng, G.Y., Gan, Y.M., Sun, Y.Y., Chao, L.I., and Zhang, X.H. (2011). ANALYSIS OF OIL CONTENT AND GC-MS OF SEEDS OF FOUR WILD PLANTS IN ABA. *Journal of Henan University of Technology*.

Wang, G.L., Meng, Q.X., and Fang, X. (2005). Analysis of Fatty Acids in Grape Seeds by GC-MS. *Journal of Gansu Sciences*.

Wang, J., Li, J.I., Ren, M., Yan, X., Wang, Y., and Shufeng, B.I. (2014). Physicochemical property and fatty acid composition of Nadina domestica T. seed oil. *China Oils & Fats*.

Wang, J.G. (2010). GC-MS Analysis on Fatty Acids in Parthenocissus quinquefolia Seed. *Journal of Anhui Agricultural Sciences*.

Wang, J.P., Meng, S.J., and Li, J.M. (1983). Fatty acid composition of three species of Litsea. *ACTA bOTANICA SINICA* 25.

Wang, Q., Wang, G.X., Tang, J., and Zhang, Z.F. (2012a). Comparative Analysis of Fatty Acids of Three Allium cepa L. Seeds by GC/MS. *China Condiment*.

Wang, Q.L., Jian-Guang, M.O., and Xie, Y.X. (2012b). Optimization of Supercritical CO2 Extraction of Pitaya Seed Oil by Response Surface Methodology. *Food Science* 33**,** 92-97.

Wang, R.F., Yang, X.W., Ma, C.M., Shang, M.Y., Yang, S., Wang, M.C., and Cai, S.Q. (2003). Analysis of fatty acids in the seeds of Sterculia lychnophora by GC-MS. *China Journal of Chinese Materia Medica* 28**,** 533-535.

Wang, T., and Chen, X. (1994). Fatty Acids from The Seed Oil of Rosa Roxburghii Tratt. *Journal of Guizhou University*.

Wang, Y. (2008). *Study on the chemical constituents of Semen Descurainiae.* Changchun University Of Chinese Medicine.

Wei, L.W., Li, P.I., Zu, and Feng, H. (2006). Studies on the lipin components in the seeds of Trigonella foenum-graecum. *Chinese Journal of Analysis Laboratory*.

Wei, M., and Liao, C.H. (2011). Component analysis of oil in acer truncatum bunge kernel in Mianyang. *Science & Technology of Food Industry* 32**,** 127-128.

Wen-Feng, L.I., Wang, X.W., Liu, F., Dai, H., Fang, G.S., and Ming, J. (2011). Analysis of fatty acids in hemp seed oil using three different esterification methods by GC-MS. *Science & Technology of Food Industry* 32**,** 120-122.

Wu Yu, H.M.-Y., Zhang, Feng-Liang, Mao Chang-Li (2015). Population Variation of Fatty Acids from the Seeds Wild Oil-bearing Tree Scleropyrum wallichianum in Yunnan. *Journal of Northwest Forestry University* 30.

Xiangyu Sun, G.G., Bo Yan, Jinmei Zhao, Liujie Gu, Jirui Liu (2012). Fatty acids and amio acids analysis of Akebia trifoliata ( Thunb. ) Koidz and Decaisnea fargesii Franch. seeds. *Journal of Chinese Medicinal Materials* 35.

Xiao Wang, C.C., Xiaolai Ma, Fuying Dong, Jianhua Liu (2002). GC-MS analysis of fatty acids from *Cucurbita moschata* Duch seed oil. *Food Science* 23.

Xin, X.L., Chen, L., Di, W.U., and Wang, Y.H. (2011). Analysis of Fatty Acid Compositions in Red Raspberry Seed Oil. *Food Research & Development*.

Xing, H.X. (2012). GC-MS Analysis on Artocarpus heterophyllus Seeds Fatty Acid Components. *Journal of Anhui Agricultural Sciences*.

Xing, W.H., Chengjiang, R., and He, L. (2010). Oil contents and relative components of fatty acid in the seeds of five energy plants. *Renewable Energy Resources***,** 62-66.

Xinghui, L.I., Fang, W.P., Zhu, X.J., Zeng, L., Luo, L.Y., and Wang, M.L. (2013). Main Chemical Composition Analysis of Tea Seed Kernel from Four Kinds of Common Tea Plant(Camellia sinensis). *Food Science*.

Yan, H.U., Ding, Y.F., Wen, C.X., Xie, X.L., Zhou, Q.M., and Liu, Y.J. (2010). Oil Contents and Fatty Acid Composition of the Seeds of the Genus Perilla from Different Geographical Origins. *Food Science* 31**,** 165-169.

Yan, H.U., Sheng, Q.Q., Zhang, T., Ding, Y.F., Wen, C.X., and Xie, X.L. (2014). Comparison of Fatty Acid Components Between Peanut Seed Oil and Perilla Frutescens Seed Oil. *Journal of Wenshan University*.

Yan, Y., He, J., Huang, X., Wang, Y., Lv, Y., and Sun, X. (2008). Physical-chemical Properties and the Fatty Acid Ingredient Analysis of Oil from Trichasanthes kirilowii. *Forest By-Product and Speciality in China*.

Yang-Min, M.A., Zhang, Q.Y., and Zhu, J.F. (2010). Analysis of fatty acids in the Chinese prickly ash seed oil by GC-MS. *Cereals & Oils*.

Yang, F., Hui, F.U., Yang, D., Zhao, N., Liu, X., and Dong, W. (2009). Physicochemical properties and chemical composition of Viburnum cylindricum seed oil. *China Oils & Fats* 34**,** 74-77.

Yang, J., Qinghua, L.I., Juan, L.I., Ping, Y.I., Na, W.U., Wang, Z., and Liu, W. (2013). Fatty acid composition of Plukenetia volubilisLinneo seeds oil. *China Oils & Fats* 38**,** 88-90.

Yang, T., Zhang, S., Yang, M., and Wu, M. (2000). Analysis of fatty acids in Foeniculum vulgare Mill. seeds by gas chromatography-mass spectrometry. *Journal of Ningxia University* 21.

Yang, Z., Zheng, M.Y., and Wei, Y.S. (2006). Study on fatty acid composition in Jasminum nudiflorum Lindl.by GC/MS. *Applied Chemical Industry*.

Yaonian Xin, B.Z., Jing Li, Yuanqi Bao, Yalin Xue (1996). Research on the biochemical constituents from seeds and oil of different Hippophae rhamnoides species. *HIPPOPHAE* 9.

Ye, C., Cui, Y., Wei, C., Elston, R.C., Zhu, J., and Lu, Q. (2011). Oil Content and Fatty Acid Composition of Camellia oleifera Seed in Guangxi. *Human Heredity* 71**,** 161-170.

Yin, S.W., Wang, W., Duan, X.J., Mao, K.J., and Guo, D. (2014). ANALYSIS OF THE NUTRIENT COMPONENTS OF THE SEED OF TUTCHERIA CHAMPIONI NAKAI. *Journal of Jinggangshan University*.

Yiping, J. (1998). Determination of fatty acids in oenathera odorata jacq seed oil by GC/MS. *FUJIAN ANALYSIS & TESTING* 7.

Yu, H., Zhang, T., Wei, C., and Li, Z. (2015). Fat contents and fatty acid composition in the seeds of three species of Suaeda. *Hunan Forestry Science & Technology* 25**,** 2077-2082.

Yu Xue-Jian, W.H.-Y., Zhang Ji-Yao, Liu Bai-Nian, Yu Qi-Tao, Huang Zhi-Heng (1991). Structural determination of fatty acid comonents in the seed oils of five species of Euphorbiaceae. *Acta Botanica Sinica* 33**,** 199-205.

Yuping Ding, Z.C., Qin Qiu, Qiang Li (2006). Supercritical fluid extraction and GC-MS analysis of *Arctium lappa* L seed oil. *Food Industry Technology* 27.

Zhang, B., Feng, F., Xin, G., and Tie-Chun, L.I. (2010). Analysis of Fatty Acids in Different Parts from Nanguoli Pear Fruits by GC-MS. *Food Science* 31**,** 410-412.

Zhang, C.L., San-Qiao, W.U., and Xin-Sheng, L.I. (2009a). Analysis on Fatty Acids Composition in Aesculus chinensis Seeds. *Seed*.

Zhang, D. (2011). Study on Chemical Components of Blackberry Seed Oil and its Antioxidant Activity. *Journal of the Chinese Cereals & Oils Association*.

Zhang, H.D., Huang, Y.L., and Han, L. (2009b). Isolation of Betel Nut Oil for GC-MS Analysis. *Food Science* 30**,** 298-300.

Zhang, H.L., Han, C.X., Wang, M.C., and Yang, Q.E. (2008). Fatty Acid Components in the Oil from Seeds of Datura stramonium L. *Acta Botanica Boreali-Occidentalia Sinica*.

Zhang, J., Tie, L.I., Wang, J., Hui, R., and Hou, D. (2004). Analysis of fatty acid in two kinds of celery seed oil by GC/MS. *China Oils & Fats*.

Zhang Jie, D.X., Shao Chengbin, Yu Yang, Liang Chong (2015a). Optimization of Supercritical CO2 Extraction of "Yanzhi" Red Radish Seed Oil and Analysis of Its Fatty Acid Composition. *Food Science* 36.

Zhang Jie, H.W., Chen Gongxi, Li Baolian, Li Cui (2015b). Fat contents and fatty acid composition in the seeds of three species of Anacardiaceae. *Hunan Forestry Science & Technology* 42.

Zhang, J.L., Tie Chun, L.I., Na, L.I., Hui, R.H., and Hou, D.Y. (2003). Analysis of Fatty Acid in Two Kinds of Pumpkin Seed Oils by GC/MS. *China Oils & Fats* 28**,** 40-41.

Zhang, J.P., Hou, X.L., Dong, H.Y., Tian, Y.U., and Zhi-Hu, M.A. (2011). Supercritical Fluid CO2 Extraction and Composition Analysis of Allium mongolicum Seed Oil. *Food Science*.

Zhang, J.S., Zhang, W.Y., Yun-Chuan, B.O., and Yao, E.M. (2007). Analysis of Fatty Acids in Almond Kernel Oil. *Acta Nutrimenta Sinica* 29**,** 308-309.

Zhang, J.X., Wan, L., Yi-Juan, H.U., Shou, Q.Y., and Yang, L.H. (2006). Analysis of Fatty Acids Composition in Semen Cassiae. *Lishizhen Medicine & Materia Medica Research*.

Zhang, J.Z. (2007). Analysis of Fatty Acids of Rosa Roxburghii Tratt Seed Oil by GC-MS. *Journal of the Chinese Cereals & Oils Association* 22**,** 85-87.

Zhang, W. (2009a). Analysis of Fatty Acids of Wild Carrot Seed Oil Extracted by Supercritical CO2 Fluid. *Journal of the Chinese Cereals & Oils Association* 24**,** 87-89.

Zhang, Z.Y. (2009b). Analysis of Fatty Acids in Alfalfa Seeds of Xinjiang. *Chinese Journal of Spectroscopy Laboratory*.

Zhao, H., Tian, G.H., and Gong, H.M. (2009). GC-MS Analysis of Fatty Acid Compositions in Fruits of Evodia rutaecarpa Benth. and E. lenticellata Huang. *Food Science*.

Zhao, Y.R., Wang, W.L., Wang, Y., Ming-Jing, L.I., and Liu, X.H. (2005). Analysis of Fatty Acid in Pomegranate Seed. *Chemical Researches*.

Zheng-Wen, Y.U., Yang, Z.N., and Yin, Y.I. (2011). Analysis on Fatty Acids of the A.annua Seeds Originated from Different Regions by GC-MS. *Seed*.

Zheng, M.Y., Ning, J.G., Geng, W., Zhang, J.C., and Wei, Y.S. (2010). The analysis on fatty components from the cherry kernel by two different esterifying methods. *Applied Chemical Industry* 39**,** 133-132.

Zhi, N.A. (2005). Fatty Acids in the Seed Oil of Calophyllum polyanthum(Guttiferae). *Journal of Tropical & Subtropical Botany* 13**,** 505-506.

Zhou, Y.H., Wang, L.S., and Wei-Guang, L.I. (2005). Analysis of physico-chemical properties and fatty acid composition of Semen Ziziphi Spinosae oil. *China Oils & Fats*.

Zhu, H.Y., Zhang, Q., Xia, C.L., Meng, X.Y., Bao, Y.L., Chun-Lei, Y.U., Yin, W.U., and Yu-Xin, L.I. (2009). Physicochemical property of euphorbia lathyris seed oil and the composition of its fatty acid and volatile oil. *Journal of Molecular Science* 25**,** 90-94.

Zhu, W.H., Xiang, Q.B., and Ou, H.Y. (1998). Seed oil fatty acids in the Diplopanax Hand. Mazz. and its systematic significance. *Journal of Plant Resources & Environment*.

Zong, W., and Ma, H. (2006). Supercritical Fluid Extraction of Lagerstroemia Speciosa Seed Oil and GC/MS Analysis. *Chinese Agricultural Science Bulletin*.

Zong, W., and Xia, W.S. (2004). Physicochemical properties of banaba seed oil and its fatty acid composition determined by GC/MS. *China Oils & Fats*.
